# Twin Peaks: A spatial and temporal study of twinning rates in Brazil

**DOI:** 10.1371/journal.pone.0200885

**Published:** 2018-07-20

**Authors:** Augusto César Cardoso-dos-Santos, Juliano Boquett, Marcelo Zagonel de Oliveira, Sidia Maria Callegari-Jacques, Márcia Helena Barbian, Maria Teresa Vieira Sanseverino, Ursula Matte, Lavínia Schuler-Faccini

**Affiliations:** 1 Post-graduate Program in Genetics and Molecular Biology, Department of Biosciences, Universidade Federal do Rio Grande do Sul, Porto Alegre, Rio Grande do Sul, Brazil; 2 INAGEMP ‒ Instituto Nacional de Genética Médica, Department of Biosciences, Universidade Federal do Rio Grande do Sul, Porto Alegre, Rio Grande do Sul, Brazil; 3 Department of Statistics, Institute of Mathematics and Statistics, Universidade Federal do Rio Grande do Sul, Porto Alegre, Rio Grande do Sul, Brazil; 4 Medical Genetics Service, Hospital de Clínicas de Porto Alegre, Porto Alegre, Rio Grande do Sul, Brazil; National and Kapodistrian University of Athens, GREECE

## Abstract

Twin births are an important public health issue due to health complications for both mother and children. While it is known that contemporary factors have drastically changed the epidemiology of twins in certain developed countries, in Brazil, relevant data are still scarce. Thus, we carried out a population-based study of live births in spatial and temporal dimensions using data from Brazil's Live Birth Information System, which covers the entire country. Over 41 million births registered between 2001 and 2014 were classified as singleton, twin or multiple. Twinning rates (TR) averaged 9.41 per 1,000 for the study period and a first-order autoregressive model of time-series analysis revealed a global upward trend over time; however, there were important regional differences. In fact, a Cluster and Outlier Analysis (Anselin Local Moran's I) was performed and identified clusters of high TR in an area stretching from the south of Brazil's Northeast Region to the South Region (Global Moran Index = 0.062, *P* < 0.001). Spearman's correlation coefficient and a Wilcoxon matched pairs test revealed a positive association between Human Development Index (HDI) and TRs in different scenarios, suggesting that the HDI might be an important indicator of childbearing age and assisted reproduction techniques in Brazil. Furthermore, there was a sharp increase of 26.42% in TR in women aged 45 and over during study period. The upward temporal trend in TRs is in line with recent observations from other countries, while the spatial analysis has revealed two very different realities within the same country. Our approach to TR using HDI as a proxy for underlying socioeconomic changes can be applied to other developing countries with regional inequalities resembling those found in Brazil.

## Introduction

Twin Peaks is an award-winning American TV series of the 90s created by Mark Frost and David Lynch, whose plot famously boasts high levels of mystery [1]. A similar mystery surrounds the “twinning peaks” of developing countries: while the incidence of twinning is well known for high-income countries, equivalent information is scarce for the developing world, mainly due to a lack of representative data [2,3].

Twinning is an important public health issue. It has been shown that morbidity and mortality are both higher in twin babies than singletons [4,5]. Health complications associated with twinning include reduced birth weight, preterm births and congenital defects [4,6,7]. In addition, mothers of twins are at an increased risk of diabetes, preeclampsia, postpartum depression and maternal mortality [8–10].

The twinning rate (TR) is defined as the number of twin births per 1,000 (‰) deliveries. In industrialized countries, rising maternal age and the increased use of assisted reproductive techniques (ARTs) have sent TRs on a dramatic upward trajectory [3,11,12]. TRs are thus a useful proxy for the use of ARTs, and where reliable statistics on ART use are scarce, comprehensive information on TRs can be valuable [3].

The epidemiology of twins in the Brazilian population remains understudied. Currently available data, derived mainly from the two states of São Paulo and Rio Grande do Sul, are highly discordant [13–16]. To the best of our knowledge, the only study to report a TR for the country as a whole, although very informative, is based on data from 1996 which addressed twinning across 75 low- and middle-income countries. As such, it did not consider Brazil’s territorial and socio-demographic diversity [17].

Here, we present data on twin births from 41 million live births registered in 5,565 municipalities across Brazil between 2001 and 2014. An important aim of the present paper is to characterize Brazilian TRs across the two dimensions of space and time. In addition, we aim to understand trends and variation in twinning data, and to identify factors relevant to the epidemiology of twins.

## Materials and methods

**Fig 1:**
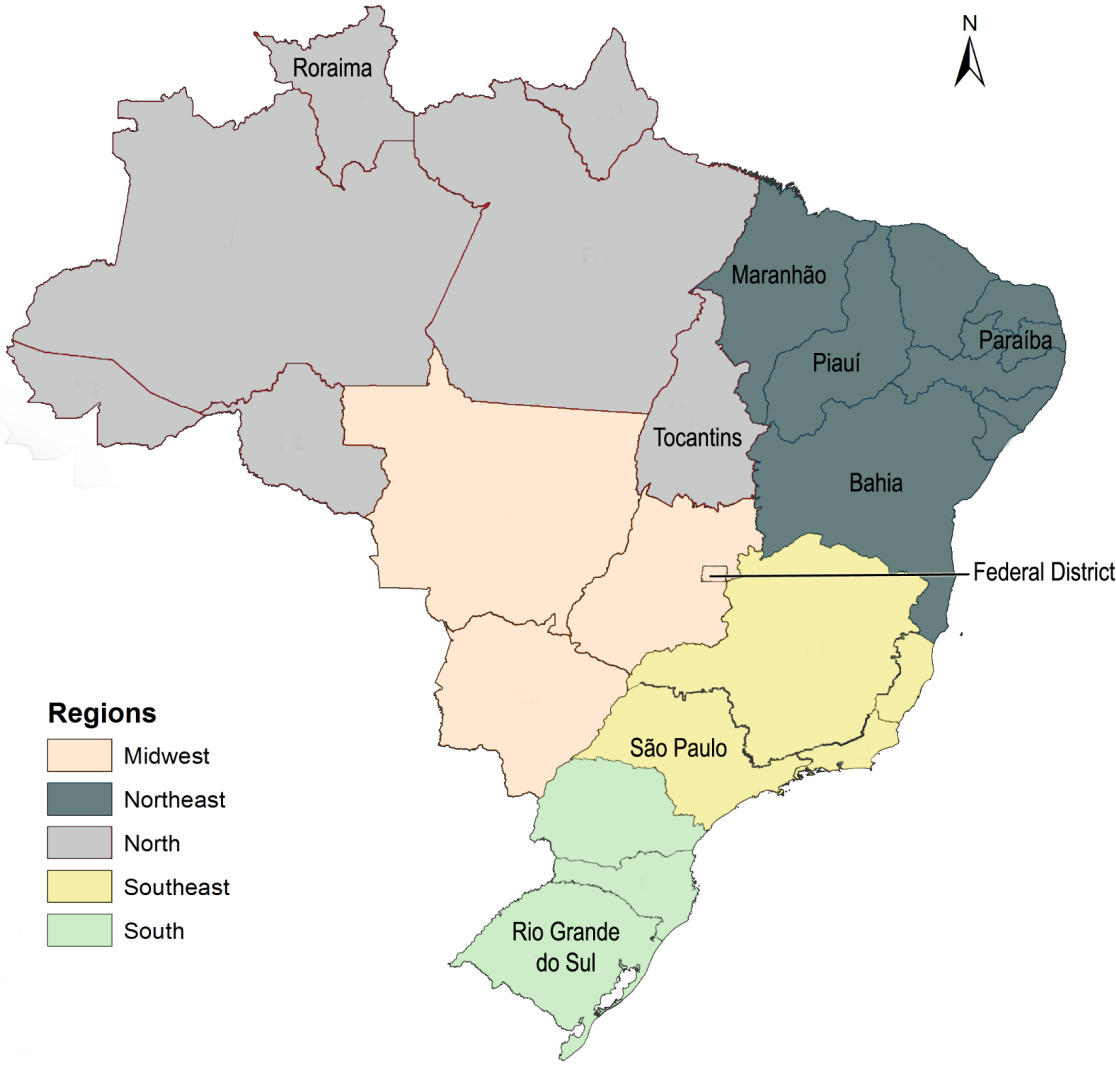
Geographical features of Brazil. The map follows the geographical division, including regions and states mentioned in the paper, of the Brazilian Institute of Geography and Statistics–IBGE (Instituto Brasileiro de Geografia e Estatística). The underlying cartographic database is publicly accessible on the IBGE website [19].

Brazil is the largest country in South America, with a population exceeding 200 million. It is divided into five geographical regions: North, Northeast, Midwest, Southeast and South (Fig 1A). The political and administrative organization of the country comprises a federal district (seat of the federal government) and 26 states (the highest administrative unit), with a total of 5,565 municipalities (the lowest administrative unit) [18,19].

### Data sources

Information on all live births registered in Brazil between 2001 and 2014 was obtained from the Brazilian Health Department’s public Live Birth Information System (SINASC). This system covers all Brazilian regions, states and municipalities [20] and has been shown to be a reliable tool for the measurement of population health indicators [21]. While SINASC provides information on singleton, twin and multiple births, it does not include stillbirths. Because twin births are associated with higher mortality than singletons [4,5], SINASC data may therefore underestimate true TRs.

TRs were calculated as follows: TR = [(individual twin births/2)/ total of deliveries) x 1,000]. Multiple birth rates were calculated using the same formula, but dividing individual multiple births by three (assuming that the majority of multiple births were triplets). We were able to analyze 41,013,511 deliveries that occurred across Brazil between January 1^st^, 2001 and December 31^st^, 2014, inclusive. Of these, 385,477 were of twins (0.94%) and 9,502 of multiples (0.02%). A total of 73,547 deliveries with an unknown type of birth were not included in our analyses.

SINASC data includes information on maternal factors linked to twinning, such as marital status, educational level and age [8,10]. Information on the gender composition of twin births, however, is not publicly available. It was therefore not possible to estimate the frequency of monozygotic (MZT) and dizygotic (DZT) twins according to Weinberg’s method [22]. Thus, like in other similar studies, the TRs reported here represent combined rates of monozygotic (MZT) and dizygotic (DZT) [3,10,12,17,23].

The use of ART-related procedures is a major factor associated with twinning peaks across the world [3,5,10,24]; however, information on ART is not recorded in the SINASC database. In Brazil, access to reproductive technologies is linked to socioeconomic factors [25–28]. As a proxy for the use of ARTs, we therefore decided to include the Human Development Index (HDI) in our analyses. The HDI is a well-established summary measure of human development, which factors in health, education, and income [18,29]. It varies between 0 and 1; the closer it is to 1, the greater the human development [30]. HDI values were obtained from the Brazilian censuses of 2000 and 2010.

### Statistical methods

To examine temporal trends for each type of birth in the country as a whole, we plotted singleton, twin and multiple rates over time. In addition, TRs were described separately for the five geographic regions, and for seven categories of maternal age at birth: ≤19, 20−24, 25−29, 30−34, 35−39, 40−44 and ≥45 years.

In our time series analysis, we used a first-order autoregressive model to identify temporal trends of TRs from 2001 to 2014. Because women over 30 are more prone to seek fertility treatment [3,25], we included a maternal age of 30 or above as a co-variable in our model. Statistically significant parameter estimates (*P* < 0.05) support the relevance of time and/or maternal age to explain the observed TRs. Time-series analysis was performed using R version 3.2.3 software [31].

Spatial analyses were conducted in ArcGis version 10.3 software using the IBGE cartographic database, which is publicly available on the IBGE website [19]. The threshold for statistical significance was set to *P* < 0.05. First, we calculated the TR for each municipality in the first (2001) and last year (2014) of our analysis, and the average TR for the analyzed time span. Second, we calculated the Global Moran Index (GMI) to test the global spatial dependence of TRs [32]. Third, we performed a Cluster and Outlier Analysis (Anselin Local Moran’s Index) to identify groups of municipalities with similar TRs or HDI (clusters). This analysis generated a map indicating statistically significant hot spots of municipalities with high TRs surrounded by other municipalities with high TRs (high-high areas), as well cold spots (low-low areas) and spatial outliers (high-low and low-high) [33]. The identification of outliers is especially useful in the case of municipalities with a high TR, which are surrounded by municipalities with low TRs. These outliers can be due to the occasional, random, occurrence of twin births against the very low background live birth rate of a small city, thus causing a substantial variation in gross TRs. Data from recently established municipalities were joined with those from the municipality from which they had emerged, in order to avoid mismatches in the analysis [34]. A similar cluster map was generated from the 2010 HDI data.

SPSS v18.0 software was used to obtain Spearman’s rank correlation coefficient (*r*_*s*_) for each individual state’s TR and HDI data. Since TR and HDI data were not consistently available for the same years, the coefficient was calculated for three different scenarios: TR data from 2001 and HDI data from 2000; TR and HDI data from 2010; average TR for the 2001–2014 period and HDI data from 2010. For the last scenario at the municipality level, we also performed a graphic Cluster and Outlier Analysis using normalized data and applying a spatial correlation index with maximum (*max*) and minimum (*min*) values of both indicators using the formula:

$$\sqrt{\frac{\left(TRmax-TR\right)}{\left(TRmax-TRmin\right)}\times \frac{\left(HDImax-HDI\right)}{\left(HDImax-HDImin\right)}}$$

Finally, we divided the municipalities into three categories according to 2010 HDI figures: low (≤ 0.599; n = 1399), medium (0.600−0.699; n = 2233) and high (≥ 0.700; n = 1933). For each category, we compared TRs from 2001 and 2014 using the Wilcoxon matched pairs test.

### Ethical approval

Because our study is based on fully anonymous, publicly available data, ethical approval was not required.

## Results

**Fig 2:**
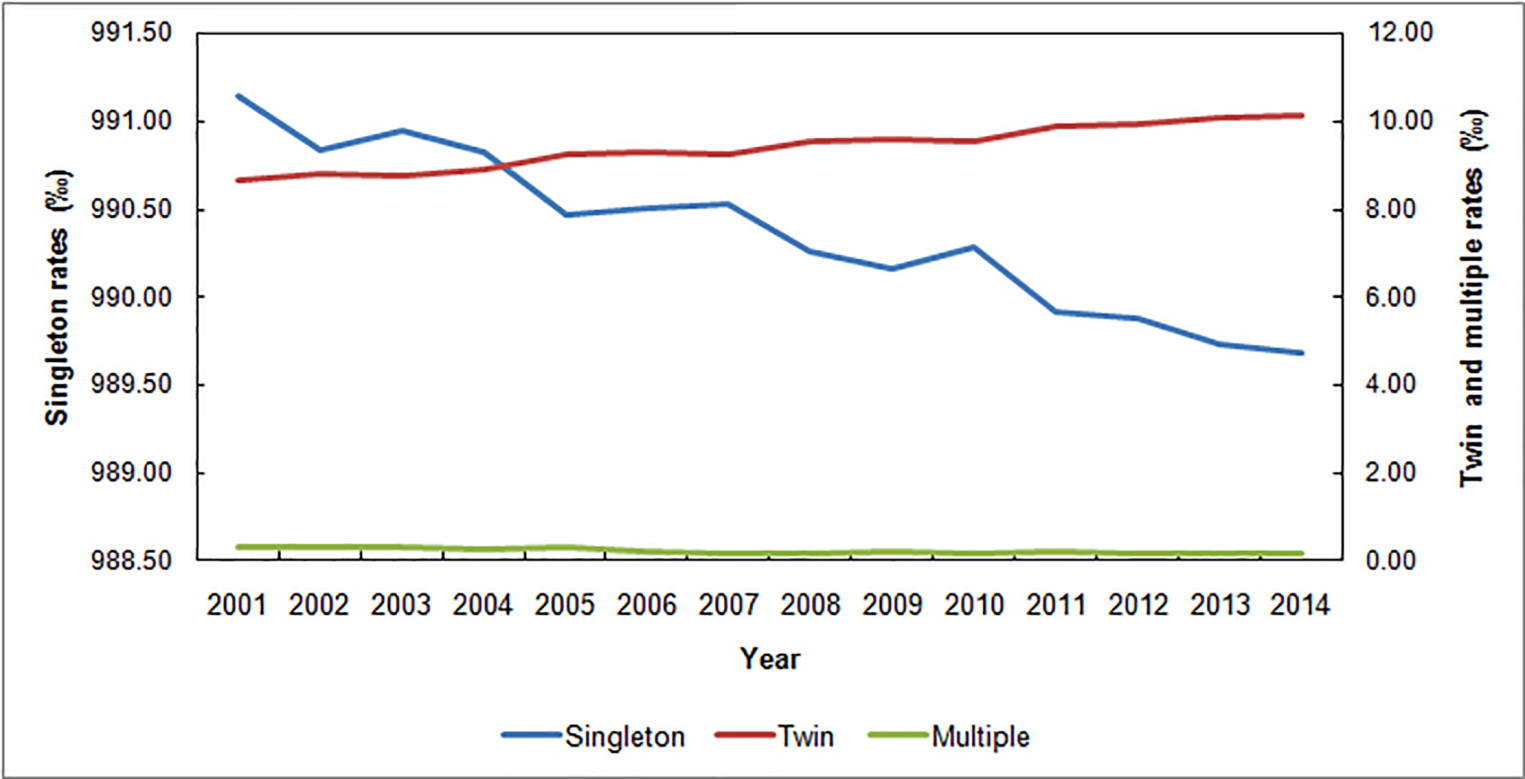
Temporal distribution. Singleton, twin and multiple birth rates per 1,000 births in Brazil, 2001–2014.

**Fig 3:**
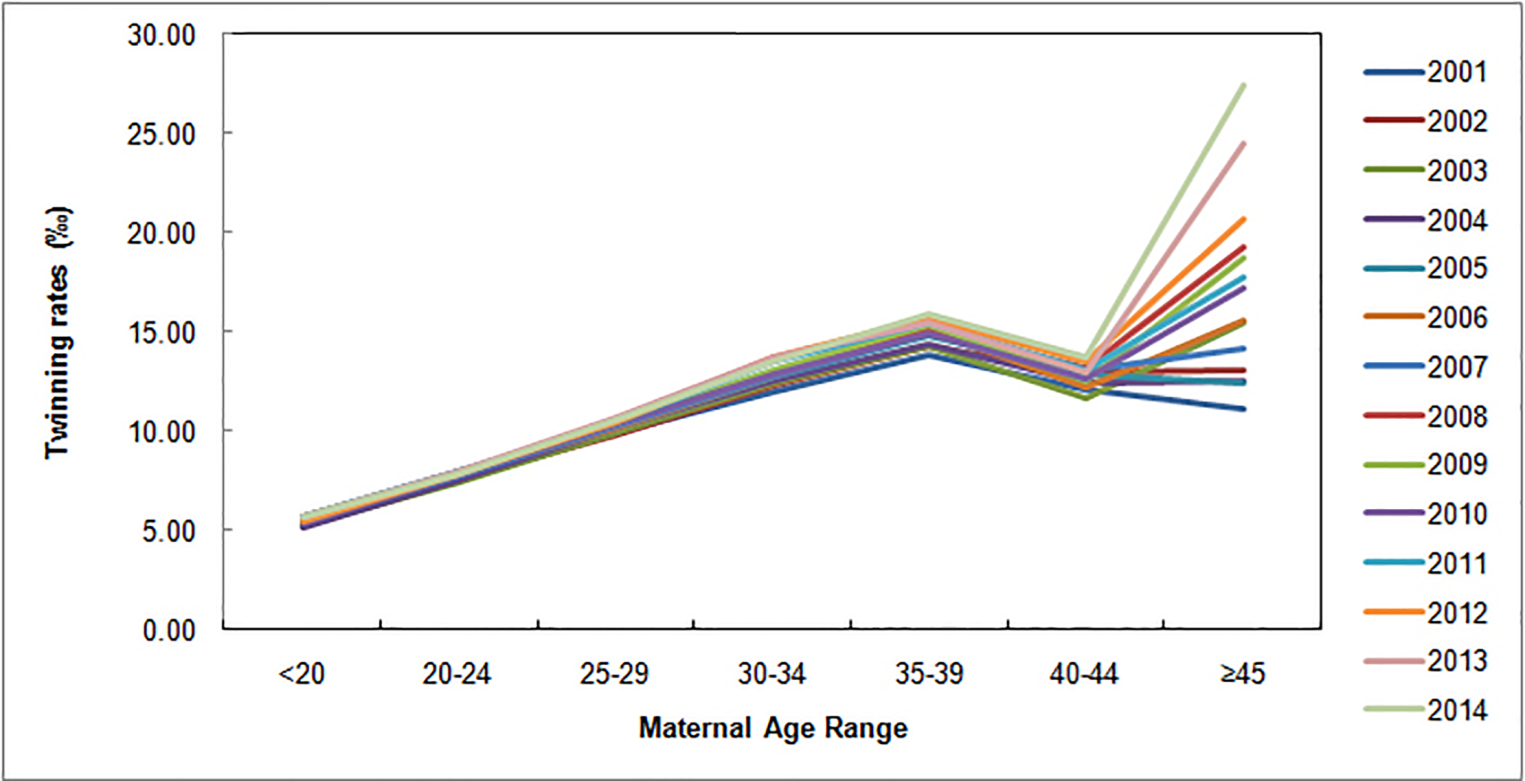
Maternal age and twinning. TRs among different classes of maternal age in Brazil, 2001–2014.

For the analyzed time period, the global average rate of twin births in Brazil was 9.41‰, or one twin birth in 106.3 live births. Over time, the average rate increased by 17.34%, from 8.65‰ in 2001 to 10.15‰ in 2014. The global average rates of singleton and multiple births were 990.37‰ and 0.23‰, respectively (Fig 2). Fig 3 presents the distribution of TRs in Brazil for the different classes of maternal age over the 14 years of the study. The increase in twinning seems to be more pronounced for women 30 years or older, and a particularly sharp increase of 26.42% was observed in women aged 45 and over.

On average, mothers of twins or multiples had completed more years of education than mothers of singletons. From 2001 to 2014, 19.77% of mothers of twins or multiples had completed 12 or more years of education (values for individual years went up from 13.79% in 2001 to 25.35% in 2014), compared to 14.86% of mothers of singletons (11.75% in 2001 to 17.36% in 2014). Similarly, the proportion of married mothers was greater among mothers of twins and multiples than among those of singletons. From 2001 to 2014, 41.84% of mothers of twins and multiples were married (45.93% in 2001 to 40.63% in 2014), compared to 35.91% of mothers of singleton (41.15% in 2001 to 32.74% in 2014).

In the autoregressive time-series model, the temporal parameter estimate was statistically significant, revealing an upward trend of TRs, both for the country as a whole and for all states of the Southeast, South and Midwest regions, as well as Bahia and Paraiba in the Northeast (Table 1). However, the temporal trend was no longer statistically significant for the whole of Brazil, as well as for most individual states, when factoring in a maternal age above 30. Instead, a positive relationship between maternal age and TR emerged, both for Brazil as a whole and for all individual states, with the exception of Roraima and Tocantins in the North, and Maranhão and Piauí in the Northeast.

**Table 1:** Brazilian twinning rates for the period from 2001 to 2014. In addition to the individual values for 2001 and 2014, the average value, percentage variation, standard deviation and the parameters estimated in the autoregressive (AR) models are also shown.

Location	Twinning rate (‰)			Variation (%)	Standard deviation	AR (temporal parameter)^a^	AR (temporal parameter)^b^	Co-variable maternal age ≥ 30^b^
	2001	2014	Average					
BRAZIL	8.65	10.15	9.41	17.34%	0.50	0.97*	0.47	0.86*
NORTH	6.92	7.54	7.31	8.88%	0.24	0.51	-0.35	0.69*
Roraima	7.68	8.47	8.17	10.22%	0.52	-0.17	-0.53*	0.54*
Acre	7.28	9.12	7.54	25.20%	0.76	0.29	0.56	1.38*
Amazonas	6.42	7.17	7.06	11.73%	0.38	0.11	-0.36	0.72*
Rondônia	7.51	6.67	6.88	-11.12%	0.98	-0.35	-0.21	0.69
Pará	6.81	7.41	7.19	8.86%	0.25	0.06	-0.21	0.62*
Amapá	7.32	6.35	7.48	-13.25%	0.71	0.02	0.16	0.59
Tocantins	7.42	8.53	7.76	14.92%	0.57	0.01	-0.57*	0.66*
NORTHEAST	8.37	9.07	8.71	8.35%	0.23	0.65*	0.04	0.55*
Maranhão	8.56	7.60	8.02	-11.17%	0.34	0.47	0.02	-1.15
Piauí	8.58	8.92	8.45	3.97%	0.58	-0.20	-0.06	0.33
Ceará	8.42	9.29	8.46	10.31%	0.35	-0.46	-0.40	0.44*
Rio Grande do Norte	8.07	9.00	8.34	11.45%	0.51	0.47	0.34	0.73*
Paraíba	8.89	9.79	8.99	10.16%	0.47	0.54*	0.03	0.83*
Pernambuco	8.37	9.11	8.83	8.84%	0.31	0.44	-0.40	0.71*
Alagoas	7.34	8.56	8.22	16.74%	0.54	0.05	-0.11	1.39*
Sergipe	8.42	9.36	9.08	11.19%	0.64	0.09	-0.02	0.81*
Bahia	8.43	9.69	9.31	14.91%	0.53	0.72*	-0.11	0.84*
SOUTHEAST	9.20	11.33	10.34	23.16%	0.73	0.96*	0.42	0.92*
Minas Gerais	9.27	10.83	10.13	16.74%	0.65	0.90*	0.45*	0.82*
Espírito Santo	8.47	10.20	9.28	20.47%	0.73	0.87*	-0.25	0.90*
Rio de Janeiro	8.88	10.43	9.91	17.45%	0.62	0.84*	0.41	0.97*
São Paulo	9.35	11.98	10.69	28.12%	0.84	0.94*	0.21	0.96*
SOUTH	9.09	11.02	10.07	21.19%	0.71	0.93*	0.05	1.17*
Paraná	9.01	10.58	9.86	17.36%	0.67	0.87*	-0.51*	1.21*
Santa Catarina	8.52	10.72	9.74	25.75%	0.79	0.81*	0.20	1.18*
Rio Grande do Sul	9.48	11.70	10.51	23.41%	0.83	0.78*	-0.18	1.12*
MIDWEST	8.31	10.17	9.07	22.39%	0.61	0.92*	-0.27	0.81*
Mato Grosso do Sul	7.96	9.93	8.73	24.82%	0.58	0.80*	0.19	1.03*
Mato Grosso	7.73	10.30	8.70	33.25%	0.83	0.81*	-0.46*	0.98*
Goiás	8.61	9.57	8.91	11.12%	0.57	0.65*	-0.72*	0.76*
Distrito Federal	8.60	11.59	10.12	34.69%	0.89	0.66*	0.48	0.83*

^a^Model considering only the time period.

^b^Model considering time and the co-variable maternal age ≥ 30.

*Stastitically significant at 0.05 level.

While an upward trend in twinning was found in all regions, there were substantial differences between regions (S1 Fig). The highest average TRs, and the highest increase in twinning over time, were observed in the Southeast region (10.39‰ average TR; 23.16% increase from 2001 to 2014). The lowest TRs were found in the North region (7.32‰ average TR, up from 6.92‰ in 2001 to 7.54‰ in 2014). Finally, the lowest percentage increase was found in the Northeast (8.35%), which also recorded the second lowest average TR (8.71‰).

**Fig 4:**
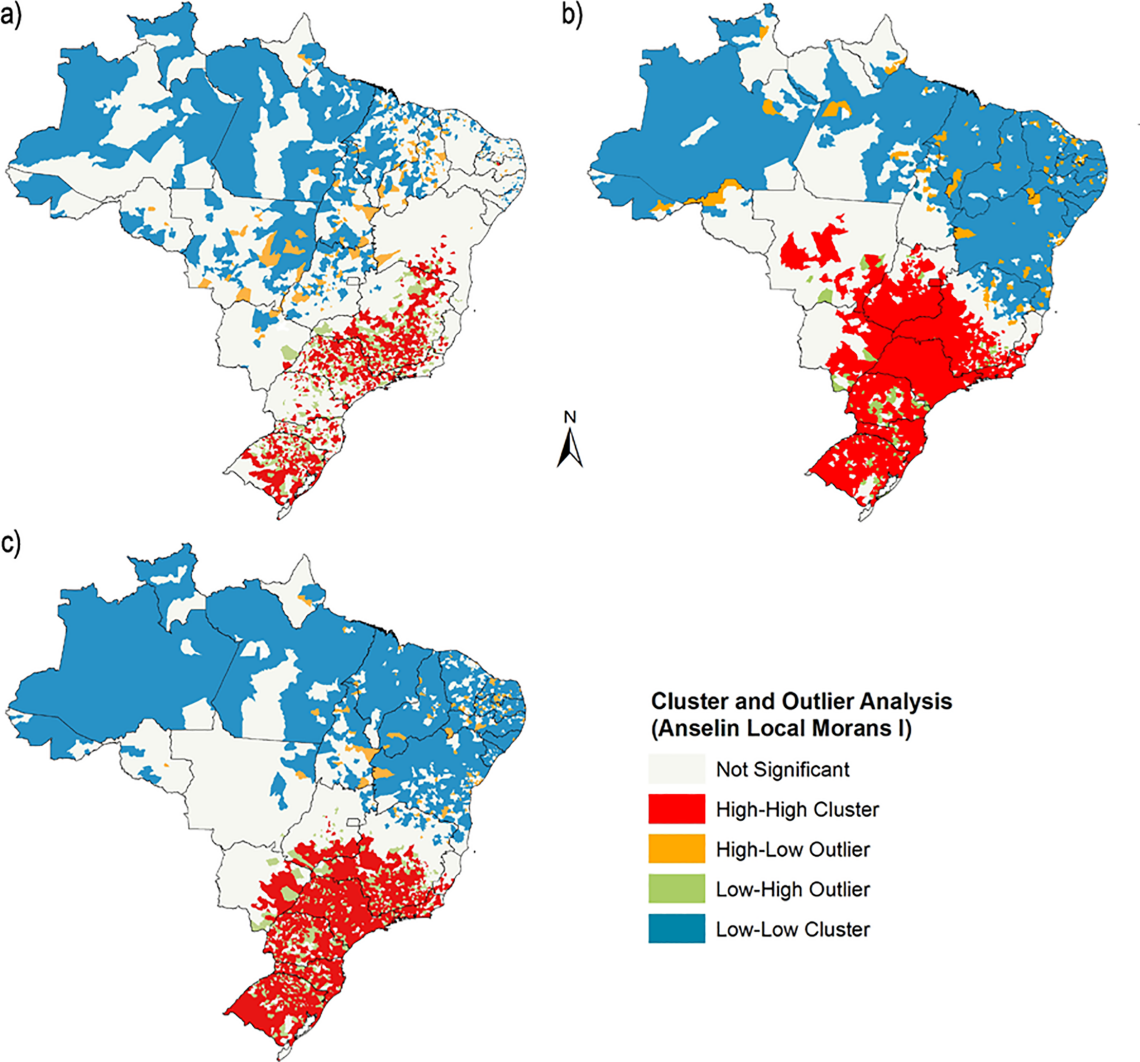
Cluster and Outlier analysis. (A) Distribution of average Twinning Rates (TRs) across Brazilian municipalities. (B) Distribution of Human Development Index (HDI) figures from the 2000 census across Brazilian municipalities. (C) Spatial correlation index from normalized average TR and 2010's HDI across Brazilian municipalities. The cartographic database used for the construction of the map is publicly accessible on the IBGE website [19].

We found a clear pattern in the spatial distribution of TRs. The GMI was 0.004 for 2001; 0.013 for 2014; and 0.062 for the average TR (all *P* < 0.001), indicating that TRs are spatially dependent in all three scenarios. From 2001 to 2014, TRs increased mainly in the municipalities of the South and Southeast (S3 Fig). A cluster of high average TRs was also observed in Paraiba, in the east of the Northeast region; however, the highest average rates were spatially concentrated in a large strip following the coastline from Bahia in the south of the Northeast region to Rio Grande do Sul in the South (Fig 4A).

The correlation analysis between TRs and HDI for each state revealed a statistically significant association in the three different scenarios studied: TRs from 2001 and HDI figures from the 2000 census (*r*_*s*_ = 0.496; *P* = 0.008); TRs and HDI figures from 2010 (*r*_*s*_ = 0.719; *P* < 0.001); and average TR for the 2001–2014 period and HDI data from 2010 (*r*_*s*_ = 0.617; *P* < 0.001). Considering this last scenario at the municipality level, the spatial correlation revealed an overlap involving both indicators (Fig 4A and 4B), with a statistically significant spatial concentration (GMI = 0.399; *P* < 0.001) (Fig 4C).

The highest TRs were observed in municipalities with a high HDI, and the lowest TRs were found in municipalities with a low HDI (S4 Fig). In municipalities with a high HDI, TRs were statistically higher in 2014 than 2001 (mean difference = 1.92; *P* < 0.001). In contrast, no statistically significant differences in TRs between 2014 and 2001 were found in municipalities where the HDI was low (mean difference = -0.74; *P* = 0.091) or medium (mean difference = 0.27; *P* = 0.331).

## Discussion

Although twin births can have important consequences for both mother and children and are considered an important public health issue, our knowledge of the epidemiology of twinning in the developing world is limited [2,17]. Here, we report an average TR of 9.41 per 1,000 deliveries for the Brazilian population between 2001 and 2014, with a peak at 10.15‰ in 2014.

The average rate reported here is slightly higher than 8.8‰, the only previously available figure for the country as a whole [17]. However, our analysis is based on a larger and more recent sample (over 41 million versus 11,099; 2001−2014 versus 1996). In addition, we present a regionally differentiated analysis, which also takes into account other factors potentially associated with twinning. Other studies have reported TRs for specific localities of the country, but the data underlying these reports are outdated and/or limited to specific regions [13,15,16].

The average rate found here for Brazil is close to that of other South American and some Asian countries, where TRs are below 9‰, and considerably lower than that of Central Africa, which has the highest incidence of twinning worldwide [5,17,35,36]. While the average rate of twinning in the Brazilian population is lower than in a number of developed countries, including the United States, France, England and Wales, and Norway, among others, the global upward trend in twinning seen in Brazil mirrors the recent trends seen in some of these countries, where increased twinning is mainly associated with two factors: delayed childbearing and/or ART [3,6,23].

Reproductive procedures, such as ovarian stimulation in hypofertile women, *in vitro* fertilization (IVF), assisted hatching and blastocyst culture, are associated with a large increase in the chance of multiple births, the vast majority of which are twin births [7,10,37]. The common practice of transferring several embryos to enhance the probability of a successful pregnancy directly increases the chances of a twin birth, despite a recent tendency to transfer fewer embryos [4,7]. However, other factors, such as the type of ovarian stimulation and the medium used in embryo culture, may also increase the incidence of MZT [38].

In Brazil, the high cost of ART represents a significant economic barrier to the use of reproductive technologies [25–28], and access to these procedures is thus generally limited to those who can afford the hefty price tag associated with them. Our results indicate that, in the absence of accurate information on ART in the Brazilian population, the positive correlation between HDI and TRs in different scenarios can help us understand the spatial and temporal distribution of twinning.

There is marked heterogeneity among the five geographical regions of Brazil in terms of demography, socioeconomics, culture, and healthcare. These differences are further accentuated by widespread internal inequalities [39]. Despite significant recent social advances, some areas in Brazil, particularly in the North and Northeast, are still characterized by a HDI typical of very poor societies. Meanwhile, the HDI of other regions, notably the Southeast and South, falls into the range of developed countries [18,30].

In a recent study, Otta et al. (2016) reported an average TR of 11.96‰ for São Paulo, the capital of the homonymous state. In our study, the highest average TRs were seen in the state of São Paulo (10.69‰) and the geographic region it belongs to, the Southeast (10.34‰), both characterized by a high HDI. Interestingly, but perhaps not surprisingly, official figures from 2014 show that the vast majority of assisted reproduction centers are located in the Southeast [40]. In stark contrast with this, in the North region, where the HDI is lower, some states have average TRs below 7‰, and no significant upward trend can be observed over the study period.

While relatively short, the 14-year period considered has been a time of enormous social and economic change across the country, as reflected by the important upward change of the national HDI from 0,612 in 2000 to 0,727 in 2010. While the indices rose more in some parts of the country than in others, they increased in all states of the country [18,29,30]. These and other changes have affected the health and health behavior of Brazilians, in addition to causing positive educational changes [39,41,42]. Taken together, such changes can result in delayed childbearing, even though some parts of the country may be affected more than others [30,42]. We found mothers of twins and multiples to have a higher educational level than singleton mothers, and increased twinning was particularly evident among older women. Especially in those aged 45 years and over, there has been a significant increase in twinning in recent years, which is likely due to the use of ART caused by difficulties in falling pregnant at an advanced maternal age.

We thus believe that the increasing TRs across Brazil, seen mainly in women over 30, as well the particularly sharp rise of twinning in women aged 45 and over, are due to a combination of five major factors: first, delayed childbearing, particularly among higher-income women [28,41,43]. Second, infertility increases with maternal age [44] and can reach high rates in developing countries [43,45]. Third, a recent (if unevenly spread) improvement in human development across Brazil [30,42]. Fourth, an increase in the availability of ART services [26,40]. Fifth, as recently emphasized by Corrêa & Loyola (2015), current ethical norms suggesting the transfer of no more than two embryos in fertility procedures amount to a recommendation, rather than an obligation or law. As a result, low standards in ART services across the country may imply an increased risk of multiple pregnancies. In addition to these major factors, an increased likelihood of spontaneous twins in older women has been recognized [10].

The democratization of access to ART in Brazil is currently a hot debate involving the scientific community, federal and state governments and the general population [25–27,43,45], and the universal access to reproductive health is one of the UN Millennium Development Goals [46]. The impact of infertility on health and well-being is undeniable, and since available treatments are currently beyond the reach of most Brazilian women [25,43], access to ART services needs to be widened. In this context, we emphasize, however, that the implementation of this public health goal requires special caution, due to the enhanced probability of twin births, which is considered to be the main iatrogenic effect of assisted medicine [7]. In some countries, TRs have declined after reaching a peak, a development which has mainly been attributed to changes in ART procedures, such as a reduction in the number of embryos transferred to the uterus [2,3].

While TRs are internationally regarded as a useful indicator of the use of medical reproductive services [3], further studies are needed to clarify the effects of ART in Brazil. The inclusion of information about ART services involved in a birth in SINASC, or even the creation of an independent database system would be very useful for such future studies.

Other factors besides maternal age and HDI as a proxy for ART may be involved in the mystery behind increased twinning, and the difficulty of analyzing them represents a limitation of this study. For example, parity has been related to twinning, and greater parity has been associated with a higher risk of twin pregnancies [5,35]. However, Brazil has achieved an intermediary stage of the demographic transition with decreasing fertility levels, making this explanation less likely [41,42]. Another factor that has been related to twin births is maternal diet, but the reports on this are conflicting [5,23,36].

The Brazilian population is one of the most ethnically admixed in the world. It has formed by extensive admixture between Europeans, Africans and Native Americans [47]. Geographically, the highest levels of European ancestry are observed in the South, while African ancestry is highest in the East, and Native American ancestry in the North [48]. Twin births are known to be linked to ethnic background and genetic predisposition [5,6,36,49]. African ancestry in particular is associated with increased twinning rates [5,17,36]. It might thus be tempting to explain our results as a reflection of genetic ancestry. However, the observed TRs were highest in the South and Southeast regions, where European ancestry is most prominent, rather than in the East, where African ancestry is particularly common. Moreover, genetic ancestry would not have changed enough over the study period to explain the upward trends in TRs observed here [3].

Our results thus indicate that the temporal and spatial distribution of TRs across Brazil are mainly linked to the effects of maternal age and ART, with local HDI figures serving as an important proxy for the socioeconomic changes underlying this scenario. Given the importance and urgency of the topic and given the lack of reliable statistics on ART across the developing world, we believe that our approach can be applied to other countries with territorial and socioeconomic profiles similar to those of Brazil, and in particular the other major emerging economies of the BRICS association.

## Supporting information

S1 Fig Twinning rates across geographical regions. Rates of twin births in Brazil and its geographical regions, 2001–2014.
(TIF)

S2 Fig Temporal distribution of births. Rates of singleton births *versus* combined rates of twin and multiple births per 1,000 births in Brazil, 2011–2014.
(TIF)

S3 Fig Spatial distribution of twinning rates. Cluster and outlier analysis showing the distribution of twinning rates across Brazilian municipalities in the years 2001 (A) and 2014 (B).
(TIF)

S4 Fig Twinning rates and HDI. Twinning rates across Brazilian municipalities grouped according to HDI categories.
(TIF)
