# Supplementary figures and images for: Twin Peaks: A spatial and temporal study of twinning rates in Brazil
Source: PLoS One. 2018 Jul 20;13(7):e0200885. doi: 10.1371/journal.pone.0200885 (PMC6054405; doi:10.1371/journal.pone.0200885)

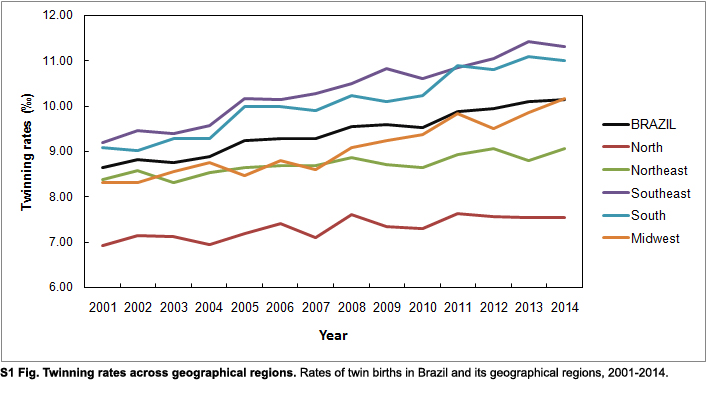

Supplement: S1 Fig — Rates of twin births in Brazil and its geographical regions, 2001–2014. (TIF) [file pone.0200885.s001.tif]

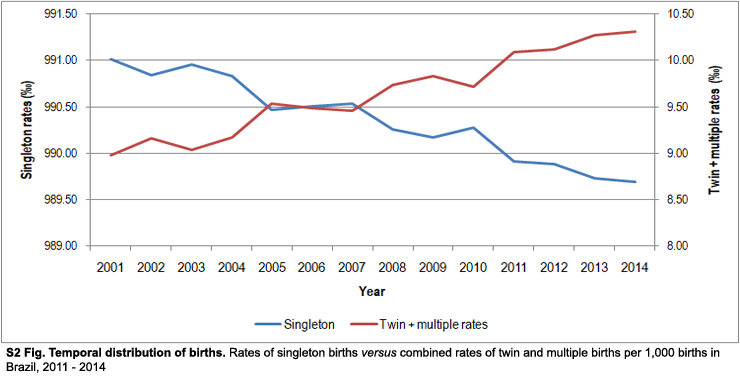

Supplement: S2 Fig — Rates of singleton births versus combined rates of twin and multiple births per 1,000 births in Brazil, 2011–2014. (TIF) [file pone.0200885.s002.tif]

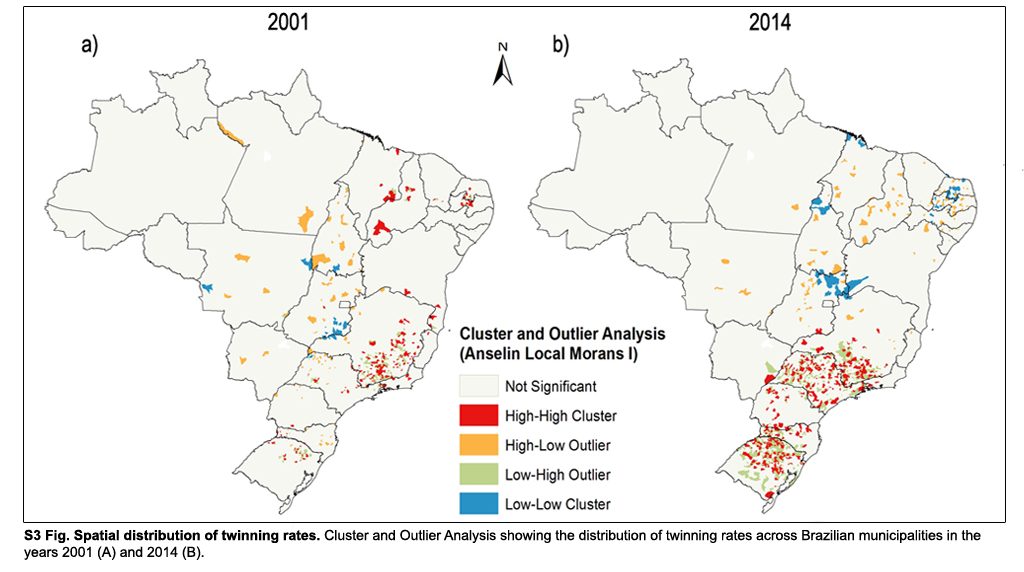

Supplement: S3 Fig — Cluster and outlier analysis showing the distribution of twinning rates across Brazilian municipalities in the years 2001 (A) and 2014 (B). (TIF) [file pone.0200885.s003.tif]

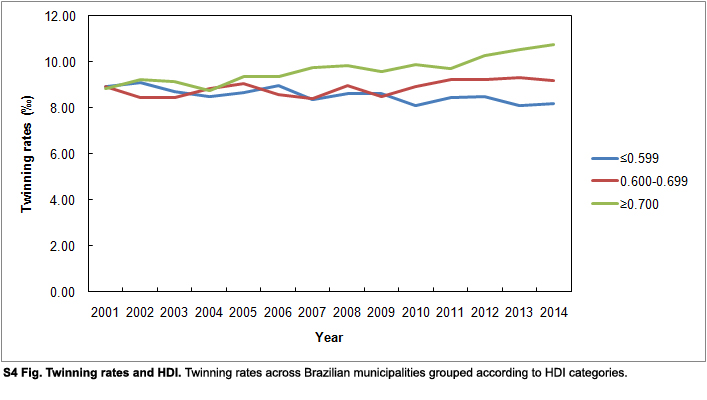

Supplement: S4 Fig — Twinning rates across Brazilian municipalities grouped according to HDI categories. (TIF) [file pone.0200885.s004.tif]
